# Perceived annoyance and asthmatic symptoms in relation to vehicle exhaust levels outside home: a cross-sectional study

**DOI:** 10.1186/1476-069X-6-29

**Published:** 2007-09-28

**Authors:** Lars Modig, Bertil Forsberg

**Affiliations:** 1 Department of Public Health and Clinical Medicine, Occupational and Environmental Medicine, Umea University, Umea, Sweden

## Abstract

**Background:**

Exhaust emissions from vehicles is a well known problem with both epidemiological and experimental studies showing increasing adverse health effects with elevating levels. Many of the studies concerning vehicle exhausts and health are focused on health outcomes where the proportion attributed to exhaust is low, while there is less information on early and more frequent subjective indicators of adverse effects.

**Methods:**

The primary aim of this study was to study perceived annoyance in relation to vehicle exhaust concentrations using modelled levels of nitrogen dioxide outside the home as an indicator with high spatial resolution. Almost 2800 persons in a random sample from three Swedish cities (Umea, Uppsala and Gothenburg) responded to our questionnaire. Questions were asked to determine the degree of annoyance related to vehicle exhausts and also the prevalence of irritating and asthmatic symptoms. Exposure was described for each participants home address by meteorological dispersion models with a 50 meter resolution.

**Results:**

We found a significant increase of peoples' self-assessed annoyance with rising levels of NO_2_. The odds of being very annoyed by vehicle exhausts increased by 14% per 1 μg/m^3 ^increase of the NO_2 _level (odds ratio (OR) = 1.14, 95% confidence interval (CI) = 1.11–1.18), and the odds of reporting the air as daily or almost daily irritating increased by 9% (OR = 1.09, 95% CI = 1.05–1.13). Also the odds of reporting asthmatic symptoms increased significantly with elevated NO_2 _levels (OR = 1.04, 95% CI = 1.01–1.07).

**Conclusion:**

This study found the degree of annoyance related to vehicle exhaust and irritating and asthmatic symptoms to be significantly dependant on the levels of traffic related pollutants outside the home. The detailed exposure assessment lowers the degree of misclassification as compared to between-city analyses, which makes the results more accurate and applicable on the local scale.

## Background

Exhaust emissions from vehicles is a well known problem with both epidemiological and experimental studies showing increasing adverse health effects with elevating levels [1,2]. Many of the studies concerning vehicle exhausts and health effects have been focused on serious health outcomes, for example respiratory diagnoses and mortality, where the proportion attributed to ambient air is low. There is less quantitative information on early and more frequent subjective measures of adverse effects. A Swedish study showed that 17% of adults in a random sample from 55 Swedish cities considered traffic exhaust fumes to be annoying, and 8% reported the air in the city centre as daily or almost daily irritating [3]. A similar Swiss study showed that approximately 18% of a random sample from 8 cities found outdoor air pollution as very annoying [4]. Annoyance is a subjective measure, and as such it's often judged less important in comparison with most physical outcomes. Nevertheless, the quantity of people being annoyed exceeds by far the number of persons with diagnosed outcomes that can be ascribed to vehicle exhaust exposure, which makes annoyance an important public health issue.

The way of assigning exposure in studies of vehicle exhausts and health vary from subjective measures to quantitative measurements of specific pollutants or dispersion modelling [5-7]. Subjective data on exposure cannot provide exposure-response functions that can be used in quantitative environmental impact assessments for roads or traffic changes. Self-assessed exposure is also a bad choice of indicator when the outcomes are self-reported, since associations may be overestimated due to reporting patterns and personality (negative affectivity) [8].

Nitrogen dioxide (NO_2_) is an important indicator of vehicle exhausts used both by local authorities when monitoring the local air pollution situation and in epidemiological studies [3,9]. Previous studies have shown significant relations between the levels of NO_2 _and the prevalence of annoyance, both with population-based and individual exposure data [3,10]. A question often raised is the comparability of results from studies using different indicators for the same exposure [4,11,12]. The within-city resolution of the exposure data is of importance to reflect the variation between subjects, which also has implications for the practical use of presented exposure-response functions in impact assessments.

The aim of this study was to study perceived annoyance and irritating and asthmatic symptoms in relation to the level of vehicle exhausts outside home as indicated by modelled levels of NO_2_. As asthmatics may suffer more from air pollutants, we also analysed doctor's diagnosed asthma as a potential modifier of exposure-response relations. Furthermore, we wanted to explore the relationship between annoyance and exposure to vehicle exhaust with more subjective, self-reported, measures of exposure in order to assess the importance of the exposure indicator.

## Methods

### Population

A random sample of 1500 persons between 16–70 years of age was drawn from the population register for each of the three cities Umeå, Uppsala and Gothenburg, giving a total sample of 4500 persons. Within the total random sample the distribution between men and woman was equal, and the mean ages of woman and men were 39 years and 38 years, respectively. In total 2766 of 4500 questionnaires were returned and possible to include in the analysis, which resulted in an overall response rate of 62%. The response rate was highest in Umea (67%) and lowest in Gothenburg (56%). Women tended to answer more frequently than men, with 54% of the respondents being women. The overall mean age was 42 years among men and 41 among women.

The cities included in the survey were chosen to represent different geographical areas of Sweden, with Umea (110000 inhabitants) located on the north east coast, Uppsala (185000 inhabitants) on the east coast and Gothenburg (490000 inhabitants) on the west coast. Within each city the sample was restricted to the central parts (suburbs excluded) based on postal code areas. To secure enough contrasts in exposure between the participants a higher inclusion probability was given to those living in the most central parts of the city. Each participant was geographically identified by the coordinates of the estate according to the home address.

### Survey

The questionnaire was sent out simultaneously to participants in all three cities in November 2004, followed by a reminder after 2 weeks and a new copy of the questionnaire after another 2–3 weeks. The questionnaire consisted of 22 questions based on the European Community Respiratory Health Survey (ECRHS), the Swedish National Environmental and Health Survey and a previous similar study conducted for the Swedish Environmental Protection Agency in 1994 [13]. Our form included questions regarding annoyance from several environmental factors, health status, the amount of traffic outside home, annoyance from traffic noise, vibrations and so forth, and questions about age, sex and housing characteristics. The survey was approved by the ethical committee at the department for Medical Research at Umeå University.

Two questions from the questionnaire were used to study how annoyed by air pollution the respondent was. The first question was "how annoying have you found pollution from road traffic close to your home during the last month", the second was "how often during winter do you find the air irritating". In the first question each participant rated their annoyance on an 11-point scale from 1 (not annoyed at all) to 11 (extremely annoyed), and in the second question the participants reported the annoyance as "daily or almost daily", "sometimes" or "seldom or never". These questions and annoyance scales have previously been used for similar assessments [3,4]. In addition the ECRHS question "Have you had asthmatic symptoms during the last 12 months (attacks or periods of shortness of breath or problems breathing)." was used since it constitutes a typical and common question on respiratory health assumed to be related to air pollution. The answer to this question was either "yes" or "no". Each participant was also asked about the presence of doctor's diagnosed asthma, rhinitis/allergy, high blood pressure and diabetes.

### Exposure

#### Modelled exposure

Meteorological dispersion models were used to calculate yearly average and winter half-year means (October through March) of NO_2 _within each city. The basis for these models is information regarding meteorological data, emission sources and emission factors from different sources. This data is then combined in the model to predict the distribution and the urban background (above roof) concentration of the modelled air pollutant. Local models prepared and managed by the local authorities were used for Gothenburg and Uppsala, whilst for Umea an external model had to be used (Gothenburg-Enviman[14], Uppsala-Airviro[15], Umea-TAPM [16,17]). For each city the mean concentration of NO_2 _was modelled in 50 meter squares. Each model was evaluated and fine tuned based on local measurements. For Umea the modelled levels of NO_2 _had to be corrected upwards by us to correspond to the levels of measured values. This is due to the complicated meteorological situation in Umea during winter because many days have very low mixing heights and inversions. The models in Uppsala and Gothenburg have recently been evaluated within a Swedish project, showing a high correlation between modelled and measured NO_2 _values [18,19].

The modelled values were combined with the coordinates representing each participant's home, giving each participant a modelled concentration of NO_2_.

A previous Swedish study used the difference in levels between cities expressed as winter half-year means to evaluate the associations [3]. Most air pollution measurements that can be used for validation have been done during the winter half-year (October-March). In this study we used both winter and yearly means.

#### Self-assessed exposure

The questionnaire included two questions that could be used as self-assessed exposure indicators to vehicle exhausts, namely; "how often do cars pass outside the kitchen window" and "how often do heavy vehicles pass outside the kitchen window". Frequency of traffic was classified as *seldom/never, often *or *constantly*.

### Statistical analysis

Multiple logistic regression was used to study the relationship between modelled levels of NO_2 _and the self-reported prevalence of annoyance and asthmatic symptoms respectively. Each annoyance question was dichotomised, where the 11 point scale was divided into less annoyed (1–8) and very annoyed (9–11) and the three category questions were divided with "*never/seldom" *and *"sometimes" *as the lower category and "*daily or almost daily" *as the higher category. The analysis was made both for the combined material and for each city separately. The city specific effect was studied by including an interaction term between city and NO_2_. All analyses were adjusted for sex, age, smoking habits, doctor's diagnosed asthma and city. Furthermore, analysis of annoyance was made using the questions of frequency of private- and heavy vehicle traffic outside the kitchen window as a self-assessed measure of exposure. This analysis was made only for the total material. The Spearman coefficient of correlation (r_s_) was used to study the co-variation between annoyance and modelled levels of NO_2_.

The probability of a person reporting him/herself as very annoyed at different levels of exposure was calculated as P(x) = 1/(1 + e^-(z)^), where x represents the level of NO_2 _and z is the linear sum expression from the logistic regression model [20]. These calculations were made for the questions "annoyed by vehicle exhausts" and "finding the air as almost daily irritating", respectively, and plotted as exposure- response curves. The relationship between the self-assessed measures of exposure and the modelled NO_2 _levels were analysed with student's t-test. Precisions in the point estimates for all analyses were estimated by calculating 95% confidence intervals (CI), with the exception of the correlation coefficient where the p-value was used.

## Results

The percentage of participants reporting road traffic close to home as very annoying is shown in Table 1. The majority rated their annoyance below 5 on the 11 graded scale, while only a small fraction (8%) of all participants was classified as very annoyed (9–11) with the dichotomised variable. Table 1 also shows the distribution within each city, with Gothenburg showing the highest percentage of highly annoyed (14%) whilst this proportion was lower in Umea and Uppsala (6%). Approximately 4% of the participants reported the air in their residential area as daily or almost daily irritating (Table 1), with the highest prevalence was seen in Gothenburg (7%) and the lowest among participants in Umea (3%). Approximately half of those finding the air as daily or almost daily irritating also report themselves as highly annoyed by vehicle exhausts.

**Table 1:** Prevalence of annoyance, perceived irritation and asthmatic symptoms.

	Annoyed by exhaust fumes from road traffic^1^		Air as daily or almost daily irritating^2^		Asthmatic symptoms^3^	
	N	%	N	%	N	%

Total	217	8.0	103	3.9	328	12.0
Umea	54	5.5	25	2.7	116	11.8
Gothenburg	109	13.7	50	6.5	105	12.9
Uppsala	54	5.7	28	3.0	107	11.3

^1 ^Estimated their degree of annoyance to 9 or higher on the 1 to 11 graded scale

^2 ^Reported the air outside home as daily or almost daily irritating

^3 ^Reported asthmatic symptoms during the last 12 months

The overall prevalence of asthma was 9.5%, whilst looking at each city separately the highest prevalence was seen in Umea (12%) and the lowest in Gothenburg (8%), (Table 2). Asthmatic symptoms were reported by 12% of the total participants, with the highest prevalence in Gothenburg (13%). Approximately 23% of all participants reported rhinitis or allergy, 12% high blood pressure and 3% reported diabetes, (Table 2).

**Table 2:** Characteristics for the whole study population and for each city separately.

	Age		Sex	Smokers	Asthma	Rhinitis/allergy	High blood pressure	Diabetes
	Mean	Min-max^a^	Men (%)	%	%	%	%	%

Total	41	16–71	54.2	20.3	9.5	23.1	11.6	3.1
Umea	43	16–70	44.9	16.7	12.3	21.1	15.6	3.7
Gothenburg	40	16–71	50.4	25.4	7.9	23.0	9.9	2.9
Uppsala	40	16–70	42.9	19.5	8.0	25.0	8.9	2.5

^a ^Minimum and maximum value

The levels and range of exposure, indicated by NO_2_, differed somewhat between the cities with Gothenburg showing the highest levels and largest Inter Quartile Range (IQR), while the levels in Umea and Uppsala were lower and within a smaller range (Table 3).

**Table 3:** Average measured^1 ^and modelled^2 ^winter mean levels of NO_2_, presented separately for each city.

	Umea	Uppsala	Gothenburg
Modelled level at central monitoring site (μg/m^3^)	20.3	17.6	28.5
			
Measured level at central monitoring site (μg/m^3^)			
2001–2002	NA^a^	13.0	27.3
2002–2003	22.8	17.0	31.8
2003–2004	18.1	15.0	27.1
2004–2005	21.7	15.3	27.7
			
Modelled levels of NO_2 _(μg/m^3^) at each study subjects home address (IQR^b^, *Min-Max*^c^)	16.9 (4.4) (*11.5–24.9*)	16.2 (2.8) (*9.6–25.9*)	29.7 (7.7) (*16.5–53.9*)

^a^NA = not applicable: no measurements available; ^b^IQR = Inter Quartile Range; ^c^Minimum and maximum value.

^1 ^Urban background levels of NO_2 _measured at a central monitoring station within each city.

^2 ^Calculated with a meteorological dispersion model within each city.

To evaluate the results from the dispersion model we compared the modelled winter average values of NO_2 _at the urban background station within each city with measured levels during several winters at the same station, (Table 3). The measured and modelled values were relatively close in all three cities. To further investigate the situation, we looked at the correlation between the modelled values and measured levels of NO_2 _at 24 measuring stations spatially distributed throughout the central parts of Umea. The comparison showed a high spatial correlation (0.8, p < 0.001) between the measured and the modelled levels.

The independent variables included in the analysis of vehicle exhausts concentration and annoyance are summarised in Table 2. The results from the analysis of the relationship between modelled levels of NO_2 _and annoyance related to vehicle exhausts, experiencing the air as irritating and reporting asthmatic symptoms, respectively, are shown in Table 4.

**Table 4:** Results from the analysis of the relation between annoyance, asthmatic symptoms, NO_2 _and traffic flow^1^

	Highly annoyed by vehicle exhausts^2^			Air as daily or almost daily irritating^3^			Asthmatic symptoms^4^		
NO_2 _winter									

	N	OR^a^	95% CI^b^	N	OR	95% CI	N	OR	95% CI
All participants^5^	2582	1.14	1.11–1.18	2526	1.09	1.05–1.13	2610	1.04	1.01–1.07
Non-asthmatics	2336	1.15	1.12–1.19	2289	1.07	1.05–1.10	2363	1.03	0.99–1.07
Asthmatics	246	1.06	0.97–1.15	237	1.09	1.03–1.15	247	1.05	0.97–1.14
For each city^6^									
Umea	921	2.32	1.54–3.51	897	1.51	0.84–2.72	927	1.28	0.84–1.94
Gothenburg	762	2.39	1.90–3.01	742	1.82	1.36–2.44	775	1.32	0.99–1.76
Uppsala	899	1.99	1.55–2.55	887	1.43	1.03–2.00	908	1.10	0.84–1.44

Private vehicle outside the kitchen window									

	N	OR	95% CI	N	OR	95% CI	N	OR	95% CI
Seldom/never	1317	1		1299	1		1329	1	
Often	634	0.95	0.58–1.57	617	0.89	0.45–1.76	637	1.01	0.67–1.54
Constantly	601	6.28	4.44–8.89	580	4.44	2.79–7.08	610	1.38	0.93–2.04

Heavy vehicle outside the kitchen window									

	N	OR	95% CI	N	OR	95% CI	N	OR	95% CI
Seldom/never	1950	1		1919	1		1964	1	
Often	331	3.89	2.59–5.78	320	4.03	2.40–6.77	335	1.38	0.84–2.25
Constantly	278	10.5	7.38–15.02	265	6.01	3.69–9.79	282	2.38	1.52–3.73

^a^OR = odds ratio; ^b^CI = confidence interval

^1 ^Logistic regression was used to estimate OR's and CI's. All models were adjusted for city, sex, age, asthma and smoking, except the separate models for non-asthmatics and asthmatics which were not adjusted for asthma.

^2 ^Estimated their degree of annoyance to 9 or higher on a 1 to 11 graded scale

^3 ^Reported the air outside home as daily or almost daily irritating

^4 ^Reported asthmatic symptoms during the last 12 months

^5 ^OR per change of 1 μg/m^3 ^in exposure

^6 ^OR for an Inter Quartile Range (IQR) increase in exposure

As seen from Table 4, increasing levels of NO_2 _are significantly related to an increase of people reporting vehicle exhausts as very annoying (odds ratio (OR) = 1.14, 95% CI = 1.11–1.18), the air as irritating (OR = 1.09, 95% CI = 1.05–1.13) and having had asthmatic symptoms during the last 12 months (OR = 1.04, 95% CI = 1.01–1.07). The results from the city-specific analyses are also presented in Table 4. In the separate analysis for each city the relative risks are presented for an IQR change of the NO_2 _level, in order to give odds ratios reflecting the effect of a relative increase in modelled exposure. Gothenburg showed the highest odds ratios in the analysis of the two annoyance questions "annoyed by vehicle exhausts" (OR = 2.39, 95% CI = 1.90–3.01) and "finding the air as irritating" (OR = 1.82, 95% CI = 1.36–2.44) while for the question on asthmatic symptoms the effects were positive but non-significant in all three cities. The analysis was also made separately for asthmatics and non-asthmatics within the combined material, but without any significant differences in the relationship to NO_2_.

**Figure 1:**
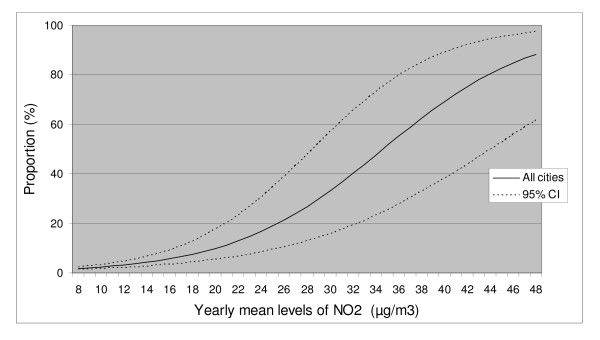
**The expected proportion of people highly annoyed from vehicle exhaust at different levels of exposure**. Highly annoyed is defined as reporting the degree of annoyance from vehicle exhausts outside home as 9 or more on a scale from 1 to 11. The exposure is based on modelled levels of NO_2 _outside each participants home.

**Figure 2:**
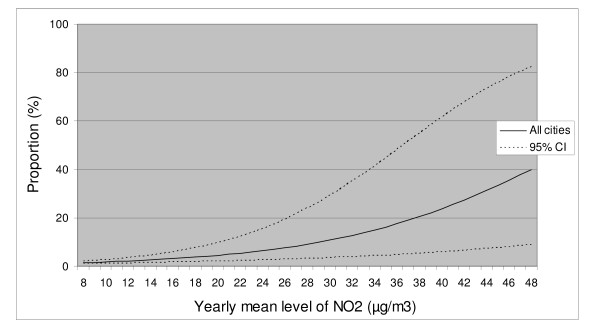
**The expected proportion of people reporting the air as irritating at different levels of exposure**. Irritating is defined as reporting the air outside home to be daily or almost daily irritating, while the exposure is based on modelled levels of levels of NO_2 _outside each participants home.

To illustrate the results as exposure-response curves we used the statistical models to calculate the relationship between the levels of average NO_2 _outside home and the probability to be very annoyed by vehicle exhausts or experiencing the air as almost daily irritating, respectively. The results are presented in figures 1 and 2.

The relationship between the three studied endpoints and the questions "How often do cars and heavy vehicles, respectively, pass outside your kitchen window" are presented in Table 4. The results show that participants reporting the frequency of private or heavy vehicle traffic passing outside their window as constantly in comparison to seldom or never were significantly more disturbed by vehicle exhausts. This was also the result for the question "finding the air irritating" whilst for the question about "asthmatic symptoms" the results were only significant in the analysis based on the frequency of heavy vehicles.

**Figure 3:**
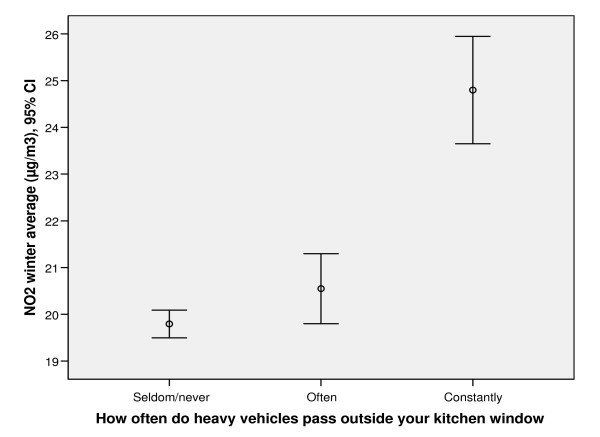
**The mean levels of modelled NO_2 _in relation to frequency of heavy traffic outside home**. Error bars showing the mean level of modelled NO_2 _outside the home within subjects reporting the frequency of heavy traffic outside the kitchen window as *seldom/never*, *often *or *constantly*, respectively.

As seen in Figure 3, people reporting that cars or heavy vehicles are constantly passing outside their home also have significantly higher levels of NO_2 _in comparison to those who report the frequency of vehicles as sometimes or seldom/never.

## Discussion

In this study approximately 8% of the total studied population reported vehicle exhausts outside home as very annoying, 4% reported the air outside home to be daily or almost daily irritating and 12% reported having had asthmatic symptoms. A study comparing different European cities regarding short-term annoyance and air pollution, showed a range in proportion of people finding air pollution as very annoying from 3% in Basel to 25% in Prague [10]. Though the severity of the outcome could be seen as somewhat less important than many health outcomes the magnitude of the prevalence makes it an obvious and important quality of life and public health issue.

The risk of being very annoyed by vehicle exhausts was estimated to an odds ratio of 3.8 per 10 μg/m^3 ^(OR = 1.14 for 1 μg/m^3^) increase in NO_2 _level, which in comparison to a previous Swiss study is approximately twice as much per μg/m^3 ^change for the same question (OR 1.8 per 10 μg/m^3^) [4]. In a Norwegian study a 10 μg/m^3 ^increase of the modelled level of NO_2 _close to home was related to a 1.6 fold increased risk of reporting high annoyance of exhaust smell [21]. The difference in size of the effect estimates between this and previous studies could be a result of differences in the selection of study population. The Swiss study included urban, rural and alpine areas while this study focused exclusively on urban areas. In the Norwegian study the selection of participants was not made to reflect a random sample but to study people before and after an infrastructural change, which makes a direct comparison more difficult. Furthermore, depending on the composition of emission sources and mainly the composition of the vehicle fleet, the degree to which NO_2 _is representative as an indicator of vehicle exhausts could differ between cities and consequently complicate the comparison of coefficients between cities. In addition, the range of NO_2 _values within each city is important when the coefficients are compared based on a given change in exposure level.

The decision to use the cut-off value 9 on the 11 graded scale to define high annoyance was based both on comparability with a previous study but also to make sure that those placed in the higher category really could be considered as highly annoyed. Lowering the cut off to 7 or 8 would only marginally impact the results as only approximately 15% of the participants reported their degree of annoyance with vehicle exhaust as above 6 on the 11 graded scale.

When studying subjective outcomes or exposures, previous studies have stressed the importance of cultural and social factors [22,23]. The impact of these types of issues together with more personal characteristics of people's perception about air pollution can consequently bias the results in epidemiological studies, especially in studies focusing on individuals and not populations. This was not a major issue when planning the study and therefore no specific questions were asked for dealing with these aspects. However, all models were adjusted for asthma, city, sex, age and smoking which to some extent are related to both social and cultural characteristics.

We also showed a weaker but significant increase in the odds for having had asthmatic symptoms during the last 12 months with rising levels of NO_2_. This question was posed to all participants, not only asthmatics, and was defined as attacks or periods of shortness of breath or problems breathing. The findings are in line with a previous study showing increasing prevalence of breathlessness during the day and urban background levels of NO_2 _[24]. A more recent study looking at respiratory symptoms among children in several countries saw no significant relation between asthmatic symptoms as *wheeze *and the levels of NO_2 _[25]. A potential explanation for the relatively weak association could be that asthmatics tend to avoid high traffic exposure. Gauderman et al showed a significant relation between asthma and wheeze and the distance to nearest freeway, while using modelled pollution from non-freeways or the traffic volume close to home did not show similar results [7]. Significant results were also seen when using measured levels of NO_2 _outside each participants home. These results suggest that it's important to describe the major source of exposure or to monitor the exposure as close to the participant as possible. In cases were there are no clear major sources, as a freeway, the resolution of the exposure data should consequently be of even more importance.

It has been shown that annoyance from vehicle exhausts often is correlated with annoyance from traffic related noise, and that noise therefore should be accounted for in these kinds of studies [21]. A Norwegian study used measures of sound pressure level (24 h LAeq) to account for noise, but in this study we did not have an objective measure for noise and therefore a similar adjustment could not be made.

Dispersion models have been used to describe exposure in previous epidemiological studies [5,7,21]. The more general comparison made within this article showed a satisfying agreement between modelled and measured values in all three cities, and a high spatial correlation in the separate comparison made for Umea. A more detailed exposure assessment gives a truer picture of people's levels of exposure which is a requirement for reliable estimates of the relationship between exposure and outcome. This has been shown in prior studies, where ambient levels of NO_2 _have been used to explain the variation in personal measurements [26,27]. The best description of a person's overall exposure to a specific pollutant is personal measurements or gathering detailed information about time activity patterns and levels of pollutants in specific micro environments. The drawback of these approaches is not only the work and costs of generating the data, but also the problem of determining which specific sources contribute to the exposure. In this study we were interested in annoyance and asthmatic symptoms as possibly related to vehicle exhaust levels at home (represented by the modelled levels of NO_2_), which is the place where most people spend most of their time. Further information regarding the modelled NO_2 _levels outside the working place and so forth would positively have contributed to give a more true total exposure assessment. However, this information was not captured by the questions used in the survey.

A population-based Swedish multi-city study showed a relatively high correlation (Pearson coefficient of correlation (r_p_) = 0.58, p < 0.01) between the urban background level of NO_2 _and the mean prevalence of experiencing the air as daily or almost daily sooty or dirty in 55 cities, while in this study the correlation coefficient for the same question was r_s _= 0.18 (p < 0.001) (r_p _= 0.22) including all participants [3]. Similar results were also seen in a study including several European cities, showing a low correlation between annoyance and personal levels of NO_2 _while a population-based comparison showed higher correlation [10]. This effect has been discussed in previous studies stating that focusing on population averages instead of individual response strengthens the correlation coefficient due to less impact from situational and person-related factors which otherwise increases the variation [28]. When it comes to describing exposure-response relationships, it is known that a more general description of traffic pollution exposure tends to increase the exposure misclassification and thereby attenuate the exposure response relationship provided that the classification error is random.

Several studies have estimated exposure-response functions for the relationship between urban background levels of air pollution and physical health outcomes, both for short- and long term exposure [29-31]. For annoyance, similar relations are official mainly for noise while for air pollution results are sparse [21,32]. In this study we present exposure-response curves for both finding vehicle exhaust close to home annoying and the air outside the home as irritating, in relation to the modelled NO_2 _average. The exposure-response functions are based on participants from all three cities which makes the relation generally applicable since the three cities differ both in size and geographical location. The main advantage with this study is, as previously mentioned, the high resolution of both exposure and individual information, which makes it possible to use the presented exposure-response relationships in local and regional health impact assessments (HIA) made with local dispersion models or local measurements.

Besides the modelled levels of NO_2_, we had information on self-reported levels of private and heavy vehicle traffic outside the kitchen window from each of the participants which could be used as a measure of exposure. Self-assessed measures of exposure and degree of annoyance have been questioned in previous studies, mainly due to insufficient evaluation against objective measures and the risk of bias when using surveys [33]. In this study it is clear that those reporting the frequency of vehicles passing outside the kitchen window as "constantly" have a significantly higher mean value of NO_2 _in comparison to those reporting less frequent traffic. Despite this fact, there are difficulties that must be interpreted and discussed before drawing any major conclusions based on subjective exposure estimates. A recent study showed that trait anxiety scores were significantly related to ratings of annoyance, which supports the use of caution when interpreting subjective exposure measures [34].

## Conclusion

This study showed that the prevalence of annoyance related to vehicle exhaust in a random sample of adults in three Swedish cities increased significantly with elevated levels of modelled NO_2 _close to the home. This effect was stronger than the effect on the 12-month prevalence of asthmatic symptoms. These associations were observed also when using more subjective measures of exposure.

## Competing interests

The author(s) declare that they have no competing interests.

## Authors' contributions

LM is the main investigator who organized the data collection, did the statistical analyses and most of the writing.

BF participated mainly in the planning and analysis and helped to write the manuscript.

All authors read and approved the final manuscript.
